# POSS with Vinyl and Epoxy Group to Enhance Dielectric and Thermal Properties of Bismaleimide–Triazine Resins

**DOI:** 10.3390/molecules30244670

**Published:** 2025-12-05

**Authors:** Wencheng Gao, Xiaoye Gao, Haosheng Wang, Riwei Xu

**Affiliations:** 1 College of Materials Science and Engineering, Beijing University of Chemical Technology, Beijing 100029, China; 2 State Key Laboratory of Mesoscience and Engineering, Institute of Process Engineering, Chinese Academy of Sciences, Beijing 100190, China

**Keywords:** bismaleimide–triazine resins, PEOVS, curing mechanism, properties

## Abstract

Bismaleimide–triazine (BT) resins are widely utilized in various applications, with ongoing efforts to enhance their performance. In this work, a partially epoxidized polyhedral oligomeric silsesquioxane (PEOVS) containing vinyl and epoxy groups was successfully synthesized, and BT/PEOVS nanocomposites were prepared by blending PEOVS with BT resin. The results revealed that the unique structure of PEOVS significantly improved its dispersion within the resin matrix and enhanced the overall properties of the BT resin. The curing mechanism and properties of BT/PEOVS nanocomposites, with weight ratios of 99.5/0.5, 99/1, 98.5/1.5, 98/2, and 96/4, were analyzed using Fourier-transform infrared spectroscopy (FTIR), differential scanning calorimetry (DSC), X-ray diffraction (XRD), dynamic mechanical analysis (DMA), thermogravimetric analysis (TGA), and dielectric measurements. The addition of PEOVS markedly improved the dielectric performance, with a 2% PEOVS content achieving a dielectric constant of 2.39 and a dielectric loss of 0.0036 at 1 MHz. Furthermore, the glass transition temperature and storage modulus were significantly enhanced, with a PEOVS content of 1.5% resulting in a glass transition temperature of 279 °C. The results demonstrate that incorporating PEOVS, featuring dual reactive functional groups, effectively enhances the comprehensive properties of BT resins, providing valuable insights into their modification and practical applications.

## 1. Introduction

The bismaleimide–triazine (BT) resins are manufactured by polymerizing 4,4′-bismaleimidodiphenylmethane (BMI) and 2,2′-bis(4-cyanatophenyl)propane (BCE). These resins have been widely used in aircraft, reinforced plastics, injection-molding powders, circuit boards, electric motor coil windings, and semiconductor encapsulation because of their excellent properties, such as high thermal stability and a low dielectric constant [1,2,3,4,5,6]. With the development of science and technology, traditional BT resins have been difficult to adapt to newer application fields, particularly given the higher requirements for their dielectric and thermal properties. Therefore, developing BT resin with improved comprehensive performance is still a field worthy of further study [7,8,9].

As a new class of organic-inorganic hybrid material, polyhedral oligomeric silsesquioxanes (POSSs) can be incorporated into a polymer matrix for preparing truly molecularly dispersed nanocomposites. They combine a hybrid inorganic-organic composition with nanosized cage structures having dimensions comparable to those of most polymeric segments or coils [10,11,12,13,14,15,16,17,18,19]. Typical POSS structures have a core unit of eight silicon atoms: (RSiO1.5)_8_, where R is a wide variety of neutral or charged organic functional groups [20,21,22,23,24,25,26,27,28,29]. The inorganic cores can enhance the resins, while the organic groups can improve the interaction between POSS and the polymer matrix. In recent years, POSS used for polymer modification has attracted much interest. Zhang et al. [30] synthesized DDSQ-type cages with difunctional and tetrafunctional linkages and copolymerized them with cyanate ester to obtain high-performance hybrid resins. Zhou et al. [31] designed and synthesized a fluorinated polyhydroxyether containing polyhedral oligomeric silsesquioxane, which was used to modify a CE resin, yielding a hybrid resin with a low dielectric constant and high impact strength. In the application of BT resin, Guo et al. [32] prepared a series of high-performance fluorinated bismaleimide–triazine (BT) resins using 2,2′-bis(4-cyanophenyl)propane (CY-1), 2′bis[4-(4-maleimidephenoxy)phenyl]hexafluoropropane (6FBMP), and diallylhexafluorobisphenol A (6FDABPA) as raw materials. The dielectric constant ranged from 2.89 to 2.94. Despite the introduction of fluorine-containing groups, the dielectric constant did not decrease significantly. Cao et al. [33] added the octa(aminophenyl)silsesquioxane (OMPS) into BT resins in order to enhance the thermal and dielectric properties of pure BT resins. Although OMPS improved the properties of BT resins, its synthesis was not easy, and the price was high. The development of high-performance BT resins with excellent dielectric properties and well-dispersed nanofillers remains a critical and promising area of research. Achieving such materials remains a significant challenge and warrants further in-depth exploration.

At present, the vast majority of research focuses on POSS with regular, single, and symmetrical functional groups [34,35,36,37]. POSS with asymmetric functional groups can act as reaction sites in composite materials to form cross-links and enhance compatibility [38,39,40,41]. Typical preparation methods for POSS with asymmetric functional groups have been reported using a fluoride rearrangement or hydrosilylation, and statistical substitution of functional groups on POSS has been reported [42,43,44,45].

This work presents the innovative synthesis of a partially epoxidized polyhedral oligomeric silsesquioxane (PEOVS) derived from vinyl POSS (OVS), enabling the simultaneous introduction of vinyl and epoxy functional groups on the POSS structure, in which epoxy groups on the POSS structure are statistically substituted [42,43,44,45]. Vinyl groups can react with imide groups, and epoxy groups can react with the -OCN groups in cyanate esters. POSS with both vinyl and epoxy groups can be introduced into bismaleimide–triazine resins to improve their dielectric properties. After incorporation into a BT resin system, the vinyl groups react effectively with BMI, while the epoxy groups form robust crosslinks with the cyanate monomer, significantly enhancing the dispersion of POSS within the BT resin matrix. Moreover, the addition of PEOVS substantially improves the electrical, dynamic mechanical, and other comprehensive properties of the BT resin.

## 2. Results and Discussion

### 2.1. Chemical Structure of PEOVS

**Figure 1:**
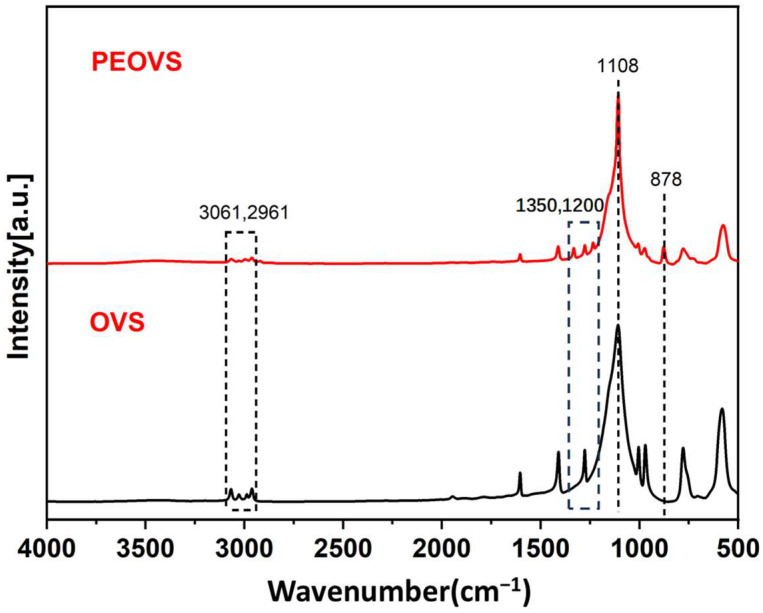
Infrared comparison of PEOVS and OVS.

Figure 1 compares the infrared spectra of PEOVS and OVS. After the reaction, a new absorption band (asymmetrical ring stretching vibration) appears at 878 cm^−1^, and a small band appears between 1100 cm^−1^ and 1300 cm^−1^, which is the absorption band (asymmetrical stretching vibration) of C–O–C, corresponding to the epoxy group. Additionally, the characteristic absorption bands (C–H stretching vibration) of the vinyl group decrease at 2961 cm^−1^ and 3061 cm^−1^, indicating partial epoxidation of the vinyl groups on OVS. Furthermore, the characteristic infrared absorption band (stretching vibration) of Si–O–Si is observed at 1108 cm^−1^.

**Figure 2:**
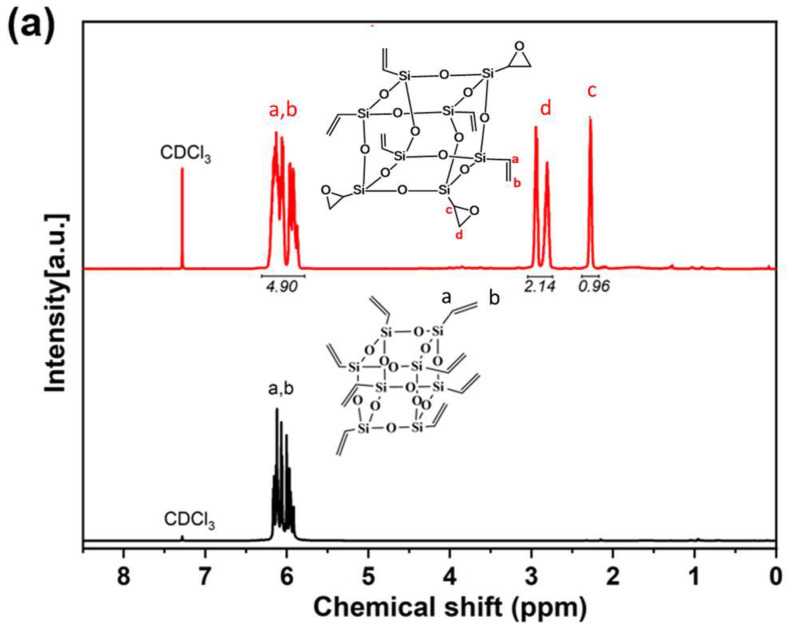

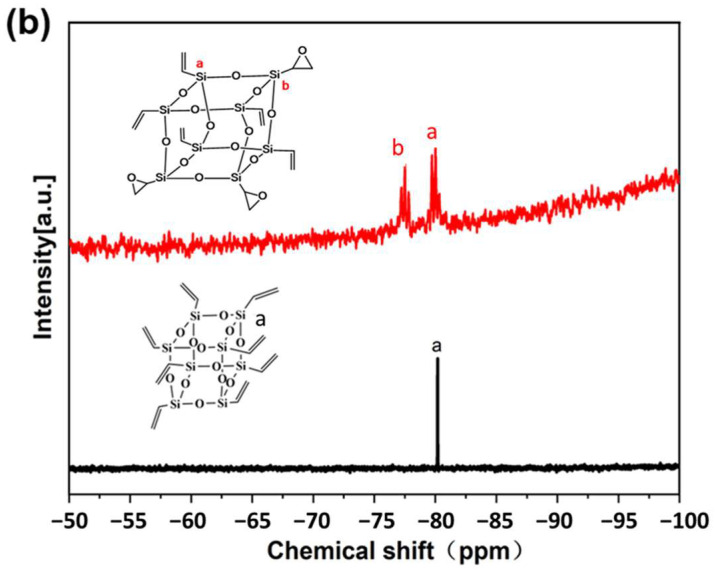
(**a**) Comparison of ^1^H NMR spectra of PEOVS and OVS; (**b**) comparison of ^29^Si NMR spectra of PEOVS and OVS.

The ^1^H NMR spectrum of PEOVS is shown in Figure 2a. The resonance lines at δ = 5.82–6.22 ppm (a, b) correspond to the protons on the vinyl group, while the resonance lines at δ = 2.77–2.97 ppm (c, d) represent the protons on the epoxy group. The resonance lines at δ = 2.27 ppm (a, b) are attributed to the protons attached to the silicon atom in the epoxy group. Based on the integration of proton signals, the average number of epoxy groups per POSS molecule is calculated to be approximately 3.1.

Figure 2b presents a comparison of the silicon spectra between PEOVS and OVS. The OVS spectrum exhibits a single type of silicon atom in its chemical environment. After the reaction, a resonance signal appears at −80.25 ppm, alongside a new resonance signal at −77.23 ppm. These results indicate the presence of two distinct silicon environments within the molecule: one associated with silicon bonded to the ethylene group and the other associated with silicon bonded to the epoxy group.

**Figure 3:**
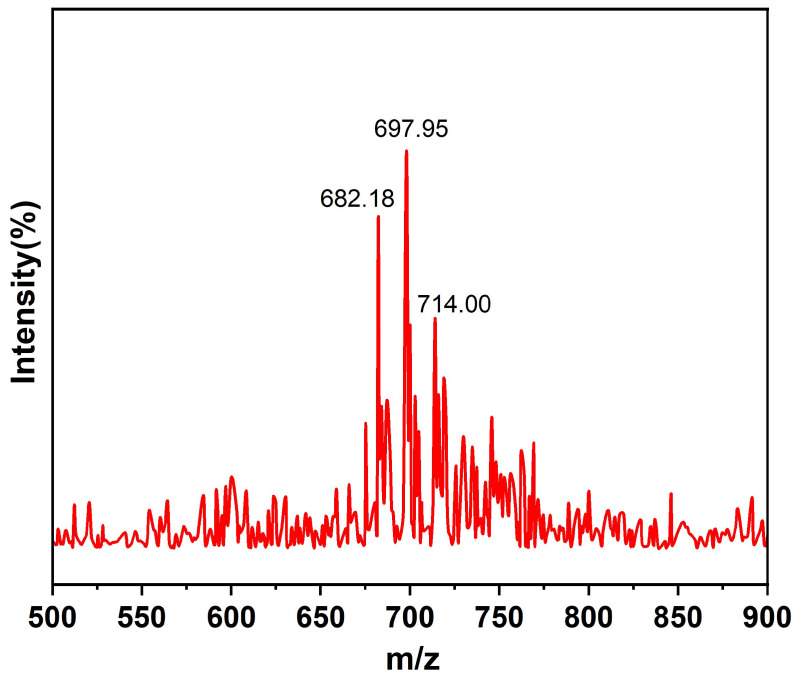
Mass spectrum of PEOVS.

The mass spectrum of PEOVS is shown in Figure 3. The spectrum reveals that the majority of POSS molecules have three epoxidized vinyl groups, consistent with the ^1^H NMR integration results. The molecular weights of POSS with 2–4 epoxy groups are M2 = 663, M3 = 679, and M4 = 695, respectively. In the mass spectrum, peaks at 682.18, 697.95, and 714.00 correspond to M3 + H_2_O, M4 + H_3_O^+^, and M5 + H_3_O^+^, respectively.

### 2.2. The Curing Behavior Analysis of Nanocomposites by DSC

**Figure 4:**
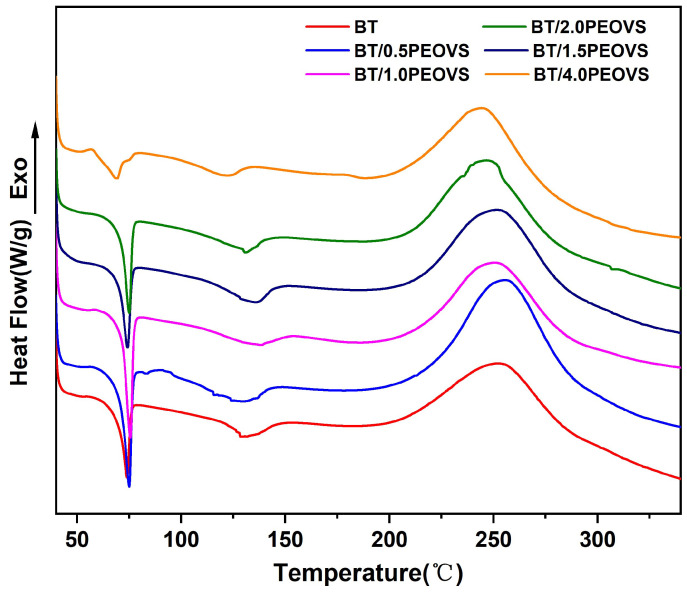
DSC curves of BT and BT/PEOVS nanocomposites with different PEOVS contents: 0%, 0.5%, 1%, 1.5%, 2.0%, 4.0%.

The thermal behavior of BT and BT/PEOVS systems is illustrated in Figure 4, and DSC parameters are listed in Table 1. The figure reveals two distinct melting peaks at 75 °C and 130 °C, corresponding to the melting characteristic peaks of cyanate ester (CE) and bismaleimide (BMI), respectively. Upon incorporating varying contents of PEOVS, no significant changes were observed in the initial curing temperature, final curing temperature, or peak curing temperature. This outcome is attributed to the low PEPOSS content. However, a notable increase in the ΔH was observed, indicating the participation of PEOVS in the formation of the crosslinked network.

Furthermore, the figure demonstrates that the peak curing temperature of the nanocomposites is approximately 250 °C. Based on these findings and previous experimental experience, the optimized curing schedule was determined as follows: 160 °C for 2 h, 180 °C for 2 h, 200 °C for 2 h, 220 °C for 2 h, and 250 °C for 2 h.

### 2.3. The Curing Reaction of BT with PEOVS

The curing system of BT/PEOVS incorporates various functional groups, including cyanate ester, maleimide, vinyl, and epoxy. To elucidate the curing mechanism of PEOVS within the BT resin matrix, the FTIR spectra of the CE/BMI/PEOVS system were recorded at different temperatures.

**Figure 5:**
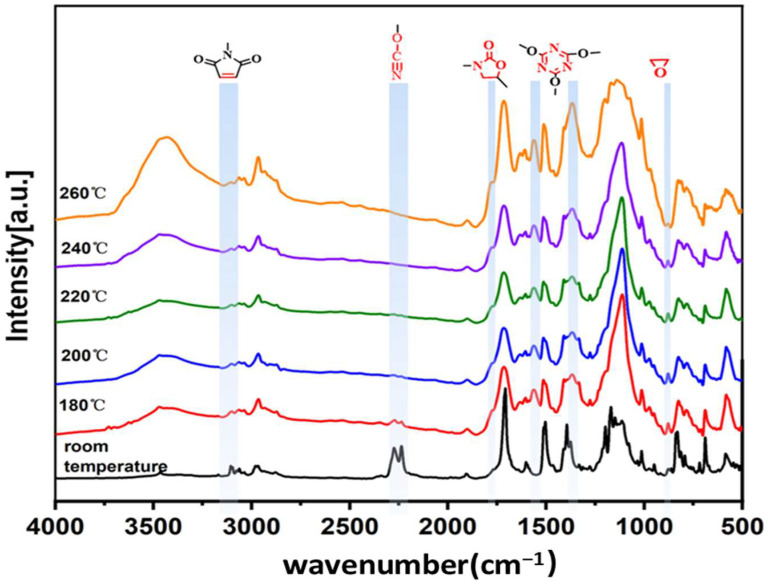
FT-IR spectra of BT/PEOVS nanocomposites at different temperatures.

Figure 5 presents the FT-IR spectra of the nanocomposites at varying temperatures. With increasing temperature, the infrared characteristic bands (stretching vibration) of -OCN at 2236 cm^−1^ and 2270 cm^−1^ gradually disappear, indicating the complete reaction of cyanate ester to form triazine rings. Correspondingly, the infrared characteristic bands (ring stretching vibration) of the triazine ring at 1379 cm^−1^ and 1564 cm^−1^ become more pronounced. Additionally, the infrared characteristic band (=C–H stretching vibration) of the BMI monomer at 3105 cm^−1^ diminishes with rising temperature, confirming the full participation of BMI in the reaction [33]. The strengthening of the infrared characteristic band (C=O stretching vibration) at 1756 cm^−1^, corresponding to oxazolidinone, and the weakening of the epoxy group band (asymmetrical ring stretching vibration) at 878 cm^−1^ on PEOVS, demonstrate the full involvement of PEOVS in the reaction.

**Figure 6:**
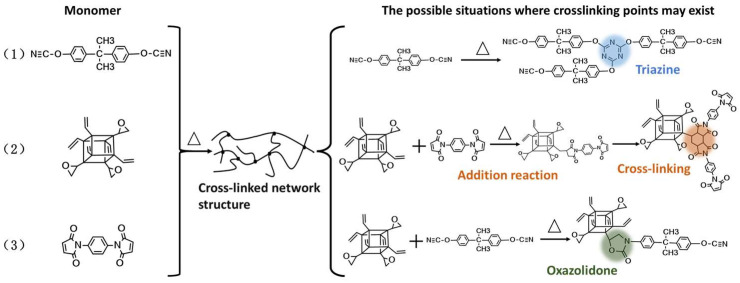
Proposed curing reactions of BT/PEOVS nanocomposites.

During the curing process, various complex reactions occur among the three components, forming a cross-linked network. According to the results in Figure 5, only the schematic diagrams of the three main crosslinking reactions are presented in Figure 6. One of the reactions is the self-polymerization of the cyanate ester monomer to form triazine rings. One of the reactions involves the polymerization of the vinyl groups on both PEOVS and the BMI monomer. The double bond in PEOVS undergoes an addition reaction with the double bond in BMI to form an intermediate, and then at a higher temperature, the double bond in the imide undergoes a Diels–Alder reaction with the intermediate to generate a polymer with a high crosslinking density. Another reaction is the formation of an oxazolidinone crosslinking network between the epoxy groups on PEOVS and the cyanate ester.

### 2.4. Dispersion of PEOVS in BT Resins

**Figure 7:**
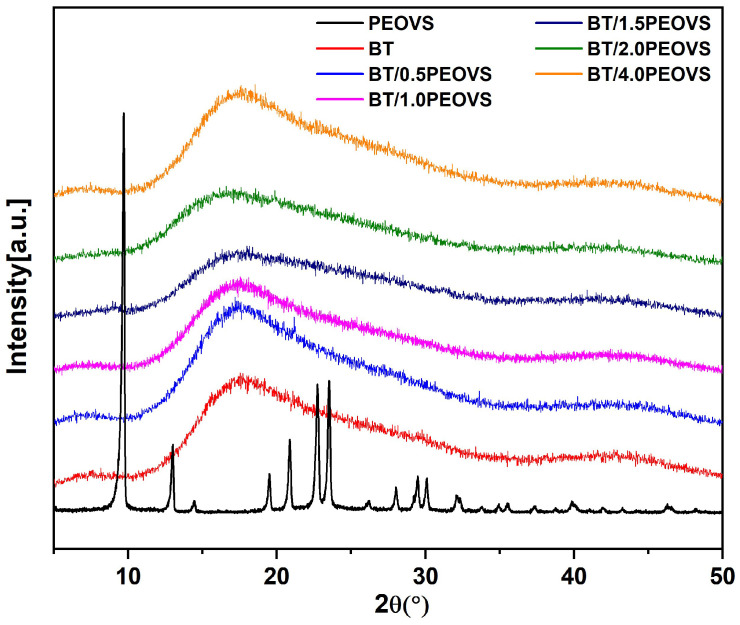
XRD spectra of PEOVS, BT, and BT/PEOVS nanocomposites with different PEOVS contents: 0.5 wt%, 1 wt%, 1.5 wt%, 2 wt%, 4 wt%.

The XRD results showed in Figure 7 that BT resins were amorphous after polymerization, while PEOVS were crystal. Both BT and BT/PEOVS nanocomposites all presented a broad disperse peak at 2θ ≈ 18°, which demonstrated that the nanocomposites were amorphous, and PEOVS dispersed very well in BT resins.

**Figure 8:**
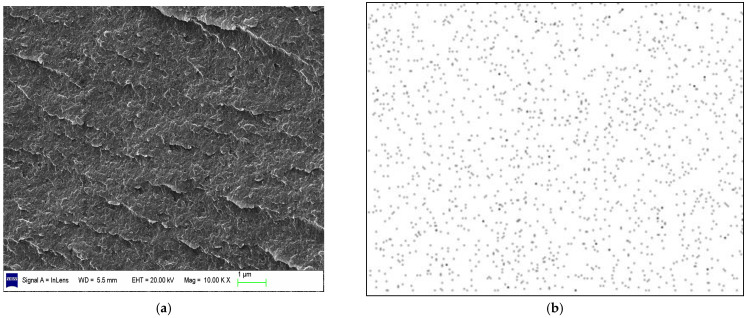
(**a**) SEM of BT/EOVS (99.5/0.5); (**b**) Si-mapping from (**a**).

**Figure 9:**
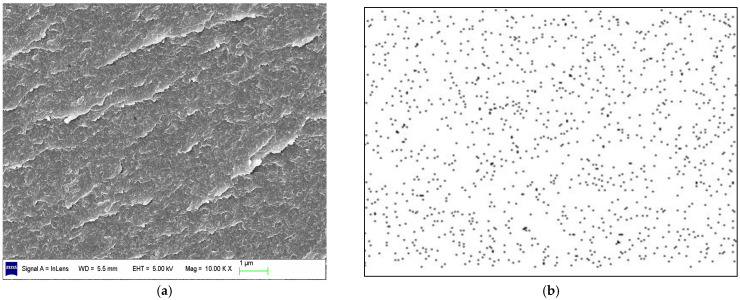
(**a**) SEM of BT/EOVS (98/2); (**b**) Si-mapping from (**a**).

**Figure 10:**
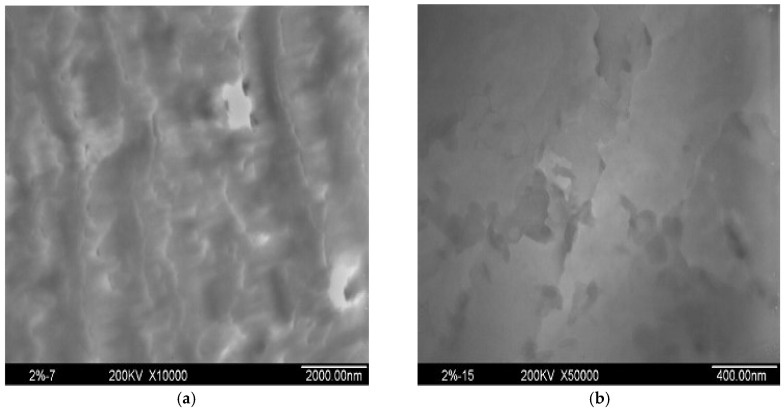
TEM of BT/EOVS (98/2): (**a**) in 2000 nm (**b**) in 400 nm.

The morphology of BT/EOVS in different mass ratios was determined using SEM (Figure 8 and Figure 9) and TEM (Figure 10). The SEM results showed that the structural context of BT/EOVS had a granular and uneven morphology. As EOVS content increased, the particles became smaller. Meanwhile, the silicon element dispersion in BT/EOVS was observed by Si-mapping, and the results showed that the silicon element in BT/EOVS was distributed equally. In the TEM photos, deep-colored particles were EOVS, while the light-colored area was the BT resin. It can be seen that the deep color particles are dispersed well in the light color area, which means that the EOVS is dispersed well in the BT resins.

### 2.5. Dynamic Mechanical Thermal Analysis

**Figure 11:**
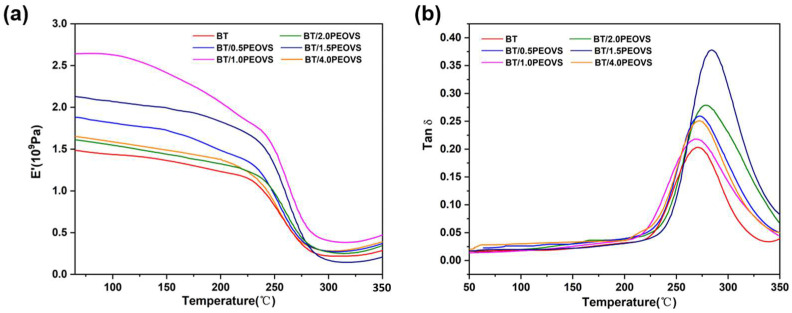
(**a**) Storage modulus (E’) of BT and BT-PEOVS nanocomposites with different PEOVS contents: 0.5%, 1%, 1.5%, 2%, 4%. (**b**) Tanδ of BT and BT/PEOVS nanocomposites in different mass ratios: 0.5%, 1%, 1.5%, 2%, 4%.

The glass transition temperature (*T_g_*) and storage modulus (E’) are critical parameters for evaluating the performance of thermosetting resins. Figure 11 shows the storage modulus and loss angle curves of the nanocomposites. The *T_g_* and E’ of the nanocomposites were investigated using dynamic mechanical analysis (DMA), and the results are presented in the accompanying figure. The E’ reflects the material’s stiffness, with higher values indicating greater rigidity and enhanced resistance to deformation.

As shown in Figure 11a, when the POSS content is 0, the initial E’ at room temperature is 1495 MPa. As the temperature increases, the E’ initially decreases gradually, followed by a sharp decline around 200 °C. With increasing POSS content, the E’ improves significantly. This enhancement is attributed to the large size effect of DEPOSS nanoparticles, which effectively integrate into the crosslinked resin network. When the PEOVS content reaches 1%, the initial E’ at room temperature increases to 2670 MPa, indicating that the formation of additional rigid network structures substantially enhances the stiffness and deformation resistance of the nanocomposite. This result underscores the unique role of PEOVS in improving the mechanical properties of the resin. Despite the low addition level of 1%, the significant improvement in E’ is closely related to the efficient crosslinking of PEOVS within the resin.

Regarding the glass transition temperature (*T_g_*), as shown in Figure 11b, it is 267 °C when the POSS content is 0. With increasing POSS content, *T_g_* gradually increases, although the improvement is relatively modest, typically within a range of 10 °C. When the POSS content reaches 1.5%, *T_g_* increases from 267 °C to 279 °C. This phenomenon is primarily attributed to the restriction of molecular chain motion caused by the incorporation of additional PEOVS, which significantly enhances the dynamic mechanical performance of the nanocomposites.

### 2.6. The Thermal Stability Analysis

**Figure 12:**
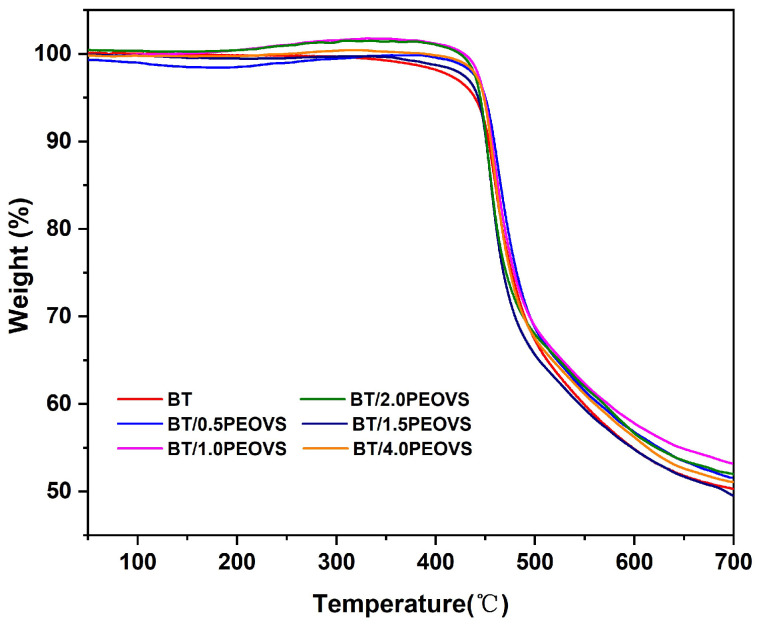
TG curves of BT and BT/PEOVS nanocomposites with different PEOVS contents: 0.5%, 1%, 1.5%, 2%, 4%.

The effect of PEOVS on the thermal stability of BT/PEOVS nanocomposites was monitored by TGA, as shown in Figure 12 and Table 2. PEOVS had a slight effect on the Td_5%_ of BT resins, and the hybrid materials maintained their thermal properties. The char yields of BT resins at 700 °C uniformly increased with the increasing contents of PEOVS, which is attributed to the good heat resistance of the Si–O–Si core in PEOVS. The tethering of the POSS cage structure to the organic polymer matrix was crucial to improve the thermal properties.

### 2.7. Dielectric Constant Analysis

**Figure 13:**
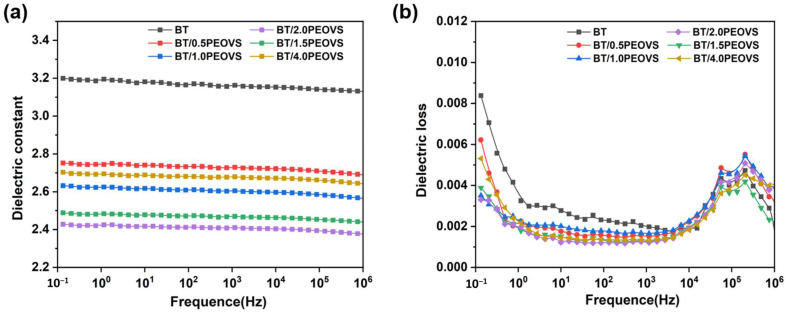
(**a**) Dielectric constant (at room temperature) of BT and BT-PEOVS nanocomposites with different PEOVS contents: 0.5%, 1%, 1.5%, 2%, 4%. (**b**) Dielectric loss (at room temperature) of BT and BT-PEOVS nanocomposites with different PEOVS contents: 0.5%, 1%, 1.5%, 2%, 4%.

As shown in Figure 13a, the dielectric constants of BT/PEOVS nanocomposites decreased slightly with increasing frequency. When the PEOVS content ranged from 0.5 to 2 wt%, the dielectric constant of the BT resin decreased. The dielectric constant can reach 2.38 at 1 MHz when the addition amount is 2%. However, as the PEOVS content exceeded 2 wt%, the dielectric constant increased again. This behavior can be attributed to the dispersion and aggregation of PEPOSS within the BT resin matrix. At lower PEOVS concentrations, uniform dispersion reduces the dielectric constant. Conversely, excessive PEOVS leads to aggregation, which increases the dielectric constant. The dielectric loss of BT/PEOVS nanocomposites remained consistently low, as illustrated in Figure 13b. Notably, the addition of PEOVS significantly reduced both the dielectric constant and dielectric loss of the BT/PEOVS nanocomposites. This phenomenon can be explained by two key factors. Firstly, during the curing process, BT resin forms triazine rings, six-membered rings, and oxazolidinone structures, all of which have low polarity and contribute to a reduction in the dielectric constant. Secondly, the incorporation of PEOVS, as a hollow cage molecule, increases the free volume within the resin matrix, thereby lowering the material density and further reducing both the dielectric constant and dielectric loss.

## 3. Materials and Methods

### 3.1. Materials

**Figure 14:**
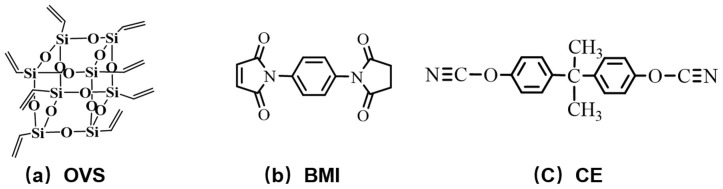
Structure of materials.

4,4-Bismaleimidodiphenylmethane (BMI) was purchased from Honghu Bismaleimide Resin Factory, Honghu, Hunan Province, China. CE was purchased from Zhejiang Kinglyuan Pharmaceutical Co., Ltd., Shaoxing, Zhejiang Province, China. Octavinylsilsesquioxane (OVS) was purchased from Shanghai McLean Biochemical Technology Co., Ltd., Shanghai, China. The other materials and reagents were purchased from Beijing Chemical Reagents Company, Beijing, China. All reagents were commercially available and were used without further purification. The structure of the main materials is shown in Figure 14.

### 3.2. Synthesis of PEOVS

**Figure 15:**
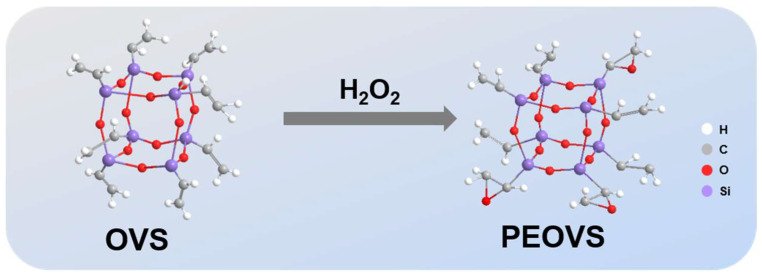
Synthetic approach of PEOVS.

The synthetic route of PEOVS is shown in Figure 15. Add 5 g of vinyl POSS, 50 mL of chloroform, 8 mL of glacial acetic acid, and 0.5 mL of concentrated sulfuric acid to a 500 mL three-mouth bottle and slowly add 6 mL of hydrogen peroxide. Condensation reflux, reaction for 6 h. After the reaction, the reaction solution was separated and washed with sodium carbonate solution and deionized water, respectively, to remove the aqueous phase. The solvent was dried in a vacuum to obtain epoxy POSS (PEOVS). The average number of epoxy groups was 3.1 per molecule.

### 3.3. Preparation of Nanocomposites

In the preparation of nanocomposites, BT/PEOVS nanocomposites (100/0, 99.5/0.5, 99/1, 98.5/1.5, 98/2, or 96/4, wt%) were made by a melting-and-blending process. The mass ratio of BMI/BCE was kept at 1:1. First, BCE resin melted at 80 °C in a 100 mL three-necked flask equipped with a reflux condenser under magnetic stirring. Then, PEOVS was added as a powder to the low-viscosity BCE resin. Finally, BMI resin was added to the flask. The temperature was increased to 100 °C and held for 20 min with stirring, until an amber, transparent, homogeneous mixture formed.

**Figure 16:**
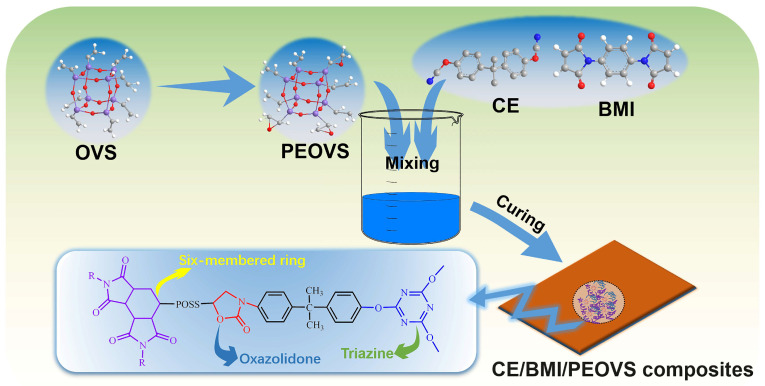
Schematic diagram of preparation of BT/PEOVS nanocomposites.

BT/PEOVS (0, 0.5, 1, 1.5, 2 or 4 wt%) were impregnated into metallic molds (40 × 8 × 2 mm^3^), degassed under vacuum at 140 °C for at least 2 h, and then cured by programmed heating as follows: 160 °C/1 h, 180 °C/1 h, 200 °C/1 h, 220 °C/1 h, 250 °C/1 h. After cooling, the samples were removed from the molds. The curing process is shown in Figure 16.

### 3.4. Characteristics of Nanocomposites

FTIR spectroscopy analysis was carried out with a Nicolet-Nexus 670 FTIR spectrometer (Thermo Fisher Scientific, Waltham, MA, USA) at room temperature (25 °C) in the range of 400–4000 cm^−1^ at a resolution of 1.0 cm^−1^. The BT/PEOVS composite samples were ground to a fine powder. Then, the powder samples were mixed with KBr and pressed into thin pellets.

DSC measurements were performed to examine the thermal behavior of BT/PEOVS nanocomposites by using a Perkin–Elmer DSC-Pyris1 differential scanning calorimetry (PerkinElmer, Waltham, MA, USA). The sample was heated at a scan rate of 10 °C/min over the temperature range from 25 °C to 350 °C with a nitrogen purge. The gas flow rate was 20 mL/min. The result was recorded with reference to empty aluminum pans.

The crystalline nature of the PEOVS, pure BT, and powder samples of the BT/PEOVS nanocomposites were measured by a Rigaku D/MAX2500 VBZ+/PC X-ray diffractometer (Rigaku, Tokyo, Japan). Cu K-α (40 kV, 200 mA) radiation was employed. The X-ray wavelength was 1.54 Å. The specimen was scanned at a rate of 5°/min within the range of 3–50°.

NMR spectra were recorded on a Bruker-AV400 MHz spectrometer (Bruker, Billerica, MA, USA) using CDCl_3_ as solvent at room temperature. Tetramethylsilane (TMS) was employed as a standard substance.

The dynamic storage modulus (E’) and loss factor (tanδ) were determined with a Rheometrics Scientific^TM^ DMTA V (Rheometric Scientific, Inc., Rochester, NY, USA) at 1 Hz. The sample was heated at a rate of 5 °C/min over the range of room temperature to 350 °C.

MS was performed on a UPLC/Premier system (Waters, Milford, MA, USA) in positive ion mode, with samples dissolved in tetrahydrofuran (THF).

A PerkinElmer TGA-7 thermal gravimetric analyzer (PerkinElmer, Waltham, MA, USA) was used to investigate the thermal stability of the nanocomposites. The samples were heated from room temperature to 700 °C at a scan rate of 10 °C/min under a nitrogen atmosphere.

Dielectric testing was recorded on an Agilent 4294A precision impedance analyzer (Agilent Technologies, Santa Clara, CA, USA) from 10^−1^ Hz to 10^6^ Hz at room temperature.

A Hitachi S-4700 scanning electron microscope (Hitachi, Chiyoda City, Japan) operating at 5 kV was applied to examine the morphologies of composites. All samples were coated with thin layers of Pt.

The Hitachi H-800 transmission electron microscope (Hitachi, Chiyoda City, Japan) was used to show the microscopic dispersion structure of EOVS in BT resin under 200 kV. The thickness of the samples was 70 nm, and they were placed on a copper grid.

## 4. Conclusions

In this work, a PEOVS with two distinct functional groups was creatively synthesized, enabling its participation in the crosslinking network of BT resin. The epoxy group in PEOVS reacts with the triazine ring, while the vinyl group undergoes an addition reaction with BMI, forming a robust crosslinking network structure throughout the system. At 1 MHz, when the POSS content is 2%, the BT resin exhibits a dielectric constant of 2.38 and an ultra-low dielectric loss of 0.0035. Furthermore, the dynamic mechanical properties and thermal stability are significantly enhanced, as evidenced by the increase in storage modulus. These findings demonstrate that polyhedral oligomeric silsesquioxanes with dual organic reactive functional groups can be uniformly dispersed in thermosetting resins, facilitating improvements in the curing process, thermal performance, and other critical properties. As research into relevant modification strategies advances, the application of resin matrix nanocomposites is expected to expand significantly.

## Figures and Tables

**Table 1:** Curing temperatures and ∆H of BT and BT/PEOVS nanocomposites in different mass ratios.

PEOVS Mass Ratio (wt%)	Initial Curing Temperature (T_i_)/°C	Peak Curing Temperature (T_p_)/°C	Enthalpy ΔH/(J/g)
0.0	183.7	253.6	249.7
0.5	177.7	253.5	470.2
1.0	182.4	251.4	404.1
1.5	175.5	252.4	319.2
2.0	179.5	247.1	299.9
4.0	182.2	245.5	277.5

**Table 2:** TGA results of BT resins and BT/PEOVS nanocomposites.

PEOVS Mass Ratio (wt%)	Temperature of 5% Weight Loss (℃)	Char Yields in 700 °C (%)
0.0	439.4	50.4
0.5	450.1	51.6
1.0	449.3	53.3
1.5	442.7	50.2
2.0	444.9	52.1
4.0	448.4	51.1

## Data Availability

Data presented in this study are available on request from the corresponding author.
